# Convolutional neural network (CNN) configuration using a learning automaton model for neonatal brain image segmentation

**DOI:** 10.1371/journal.pone.0315538

**Published:** 2025-01-17

**Authors:** Iran Sarafraz, Hamed Agahi, Azar Mahmoodzadeh

**Affiliations:** Department of Electrical Engineering, Shiraz Branch, Islamic Azad University, Shiraz, Iran; South China University of Technology, CHINA

## Abstract

CNN is considered an efficient tool in brain image segmentation. However, neonatal brain images require specific methods due to their nature and structural differences from adult brain images. Hence, it is necessary to determine the optimal structure and parameters for these models to achieve the desired results. In this article, an adaptive method for CNN automatic configuration for neonatal brain image segmentation is presented based on the encoder-decoder structure, in which the hyperparameters of this network, i.e., size, length, and width of the filter in each layer along with the type of pooling functions with a reinforcement learning approach and an LA model are determined. These LA models determine the optimal configuration for the CNN model by using DICE and ASD segmentation quality evaluation criteria, so that the segmentation quality can be maximized based on the goal criteria. The effectiveness of the proposed method has been evaluated using a database of infant MRI images and the results have been compared with previous methods. The results show that by using the proposed method, it is possible to segment NBI with higher quality and accuracy.

## 1. Introduction

Medical image segmentation is one of the main applications of image processing techniques in the field of medicine. In this field, BIS (Brain Image Segmentation) has received particular attention [1], and helps doctors in diagnosing abnormalities and complications such as tumors, tissue damage, or detecting plaques of MS (Multiple Sclerosis) [2]. These techniques’ scope of application in medical science is continuously expanding. For example, by using BIS techniques, diseases related to brain spectrum disorders, such as autism, can be identified [3]. According to this scope of application, many methods for BIS have been developed in recent years, in which NBIS (Neonatal BIS) has been of particular interest due to the fundamental differences between the structural features of the neonatal brain (NB) and adult brain (AB) [4]. Here, the term neonate refers to infants under one year of age. The main difference between NB and AB is the lack of development because the human brain grows continuously from birth to adulthood. This lack of development is more evident in the first months of the baby’s growth [5]. Thus, new challenges arise in NBIS, which may not be relevant to ABIS (AB Image Segmentation). One of these challenges is the lower contrast in NB images compared to AB due to different water level in different parts, which results in high overlap of gray levels in White Matter (WM), Gray Matter (GM), and Cerebrospinal Fluid (CSF) [6]. The second challenge is the high level of anatomic changes of NB, which is more evident in the case of diseases involving the brain. Rapid changes in NB structure and volume are challenging for NBIS, and for this reason, the NBIS process should be done independently of these changes [7].

Deep learning techniques, especially CNNs, are among the most efficient learning-based methods for image processing. Image segmentation has always been considered as one of the main applications of these learning models, and various models based on CNN structure have been presented for image segmentation [8]. Despite the high efficiency of these models, their performance still cannot be considered favorable. The structural nature of CNNs makes it possible to imagine infinite configurations for each CNN structure, and applying any change to the configuration can change its performance. For this reason, providing solutions to determine the automatic and (nearly) optimal configuration in CNNs is one of the requirements. In [9], a method based on dilation and attention mechanism was introduced for 3D NBIS. The deep learning model presented in this article is dilated attention mechanism with attention loss (DAM-AL), and it consists of two strategies: dilated attention mechanism and hard-case attention loss. DAL-AL model includes a spatial attention mechanism for high-level features and a channel-based attention mechanism for low-level features. In [10], the combination of random forest and Gabor filter bank was employed for NBIS. In this method, MRI images are pre-processed with the DM3D denoising method. In [11], a technique called IAS-NET based on deep learning was introduced for NBIS. The IAS-NET model is a learning framework that transfers the appearance features of images from two aspects of image and feature across domains. A novel 3D densely connected convolutional network architecture for brain volume segmentation is proposed in [12] that uses dense connections between layers and combines multiscale features to improve segmentation accuracy. Several deep-learning models have been presented for NBIS. Some of these models are APRNet [13], Non-local U-Net [14], and FC-Semi-DenseNet [15]. In this article, an adaptive mechanism is presented to determine the optimal configuration of CNN. This solution includes a set of LA(Learning Automaton) models (a decision-making model that iteratively learns the optimal action to take in a random environment based on the feedback received and helps network adaptively find the best architecture and parameters, such as filter sizes, pooling types, and number of layers, by employing a reward and penalty mechanism during the training), where each LA is responsible for dynamically determining the optimal value for one of the CNN hyperparameters. Then, using DICE and ASD segmentation quality evaluation criteria, the optimality of the created configuration is evaluated. In this iterative process, a set of LAs using reward and penalty operators leads the CNN configuration to the optimal state. The proposed LA-based CNN method is designed to be flexible, adaptive, and compatible, allowing for seamless integration with existing deep learning models and systems, including those used in medical imaging applications [16]. It supports common deep learning frameworks such as TensorFlow and PyTorch, which ensures straightforward integration into various research and clinical environments [17]. Additionally, the use of standard image formats and conventional segmentation quality measures ensures that it can be easily extended to other segmentation tasks beyond neonatal brain imaging. Our approach is optimized for GPU-based parallel processing, allowing it to scale efficiently with complex network architectures and large datasets. This scalability makes it suitable for integration into cloud-based medical imaging platforms, which can facilitate remote training and deployment [18]. By utilizing existing APIs, the proposed system can interact seamlessly with external software tools, ensuring smooth data handling, model training, and inference [improv].

The continuation of the article is organized as follows: in section 2, the previous related studies in the field of BIS are reviewed. In section 3, the proposed method is described. In section 4, the efficiency of the proposed method has been evaluated from different aspects. Finally, in section 5, the findings are summarized.

## 2. Proposed model for NBIS

**Fig 1:**
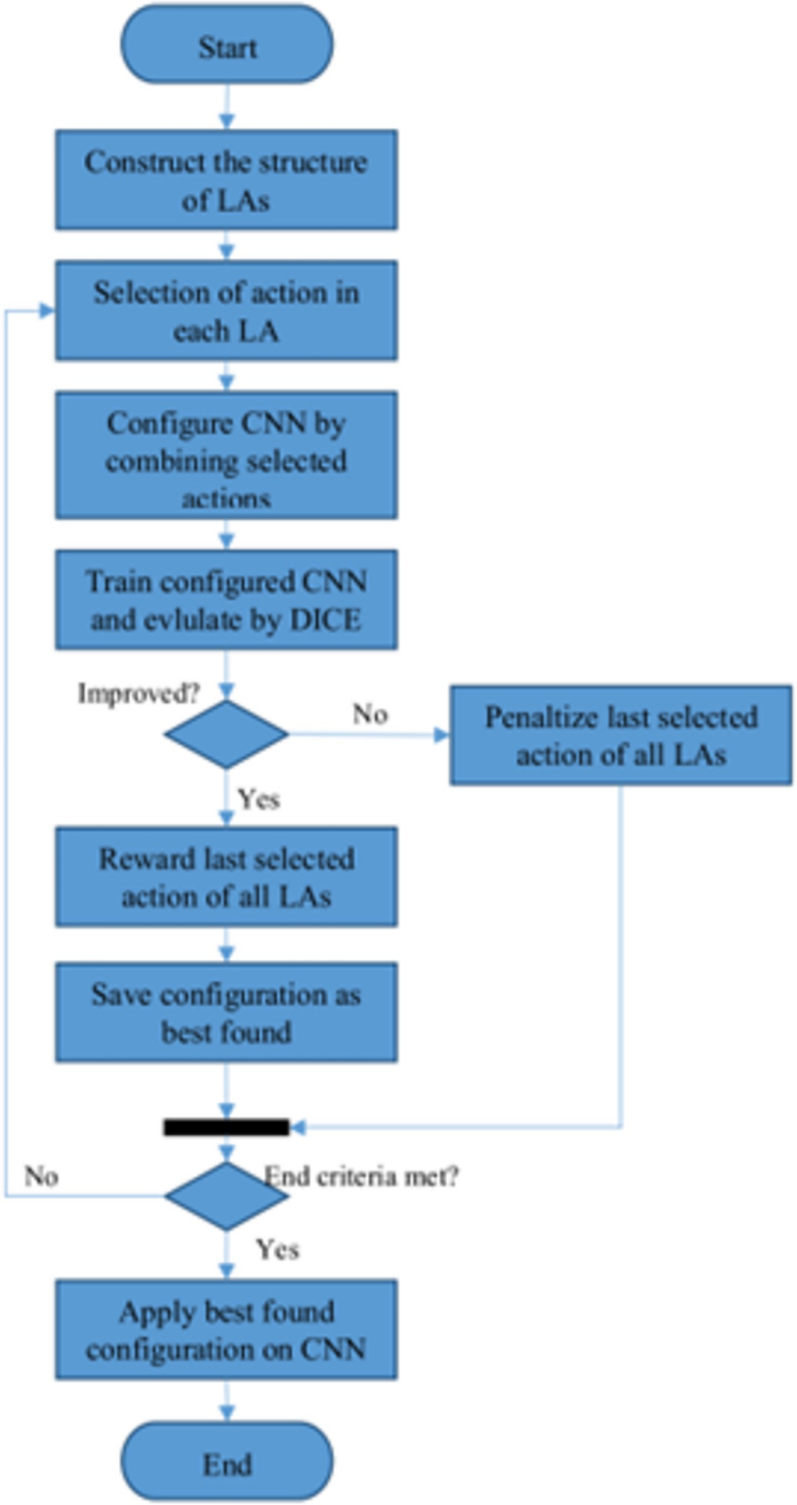
CNN automatic configuration process in the proposed method.

The proposed NBIS model is based on a basic CNN pattern whose hyperparameters are tuned by a set of LAs. As shown in Fig 1, the CNN model used in the proposed method is based on coding-decoding, in which configurable hyperparameters are dimensions and the number of filters in each convolution layer, as well as the type of pooling functions. In this way, an LA model is assigned to each configurable layer to determine the optimal combination of hyperparameters in the corresponding layer. At the end of this iterative process, a model with the highest segmentation quality is obtained.

According to Fig 1, the proposed plan begins with creating the LAs structure. In this step, an LA model is assigned to each configurable layer, and the action set of each LA is equal to all possible choices for the configuration of that layer. After the formation of these RL (Reinforcement Learning) models, each model selects an action. This choice can be made randomly or based on the probability vector created in automata, which is explained in the following sections. By combining the choices made by the LAs, a configuration for the CNN model is created. In the next step, the resulting CNN model is trained, and its segmentation quality is evaluated based on the training samples by the DICE criterion. If DICE criterion shows any improvement in segmentation quality compared to previous iterations, then the recently selected actions by the LA models are updated by the reward operator and this configuration is saved. Otherwise, the possibility of all recent actions will be reduced using the penalty operator. This process is repeated until at least one termination condition is met. The termination conditions of the proposed algorithm are:

✓ reaching the maximum number of iterations, T
✓ the quality criterion was not improved after K consecutive iterations
✓ reaching the maximum value for the quality criterion

### 2.1. The structure of the basic CNN pattern in the proposed method

**Fig 2:**
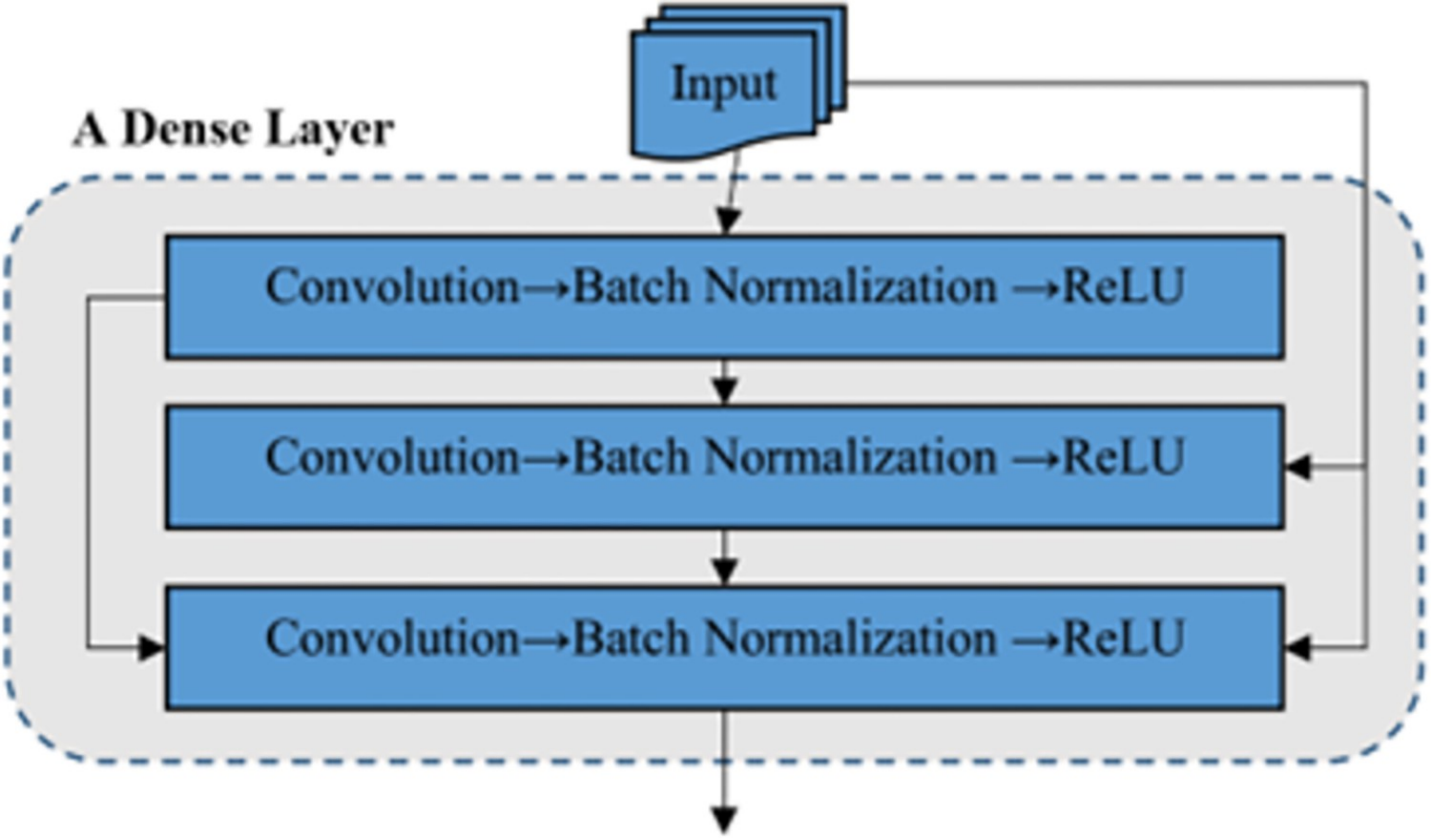
The structure of each dense layer in the proposed basic CNN pattern.

The basic CNN model in the proposed method is based on an encoder-decoder structure [19]. Thus, this model consists of two coding and decoder parts, which ends with a Softmax layer. Each of the coding and decoder parts consists of three dense layers [20]. The number of layers in both mentioned parts is equal, But the configuration of these layers is not necessarily symmetrical. Each dense layer contains three consecutive layers, consisting of convolution, normalization, and activation operators. The structure of each dense layer is shown in Fig 2.

According to Fig 2, in a dense layer, the input data is applied to all internal convolution layers, and the output of each layer is transferred to the layers after it. The activation function is determined in each inner layer of the ReLU type. Thus, for each of the internal layers in the dense layer, if the input data is *X*, then the output of this layer can be described as follows:

$$y_{l}=ReLU(BtachNorm(Conv(x)))$$ (1)

**Fig 3:**
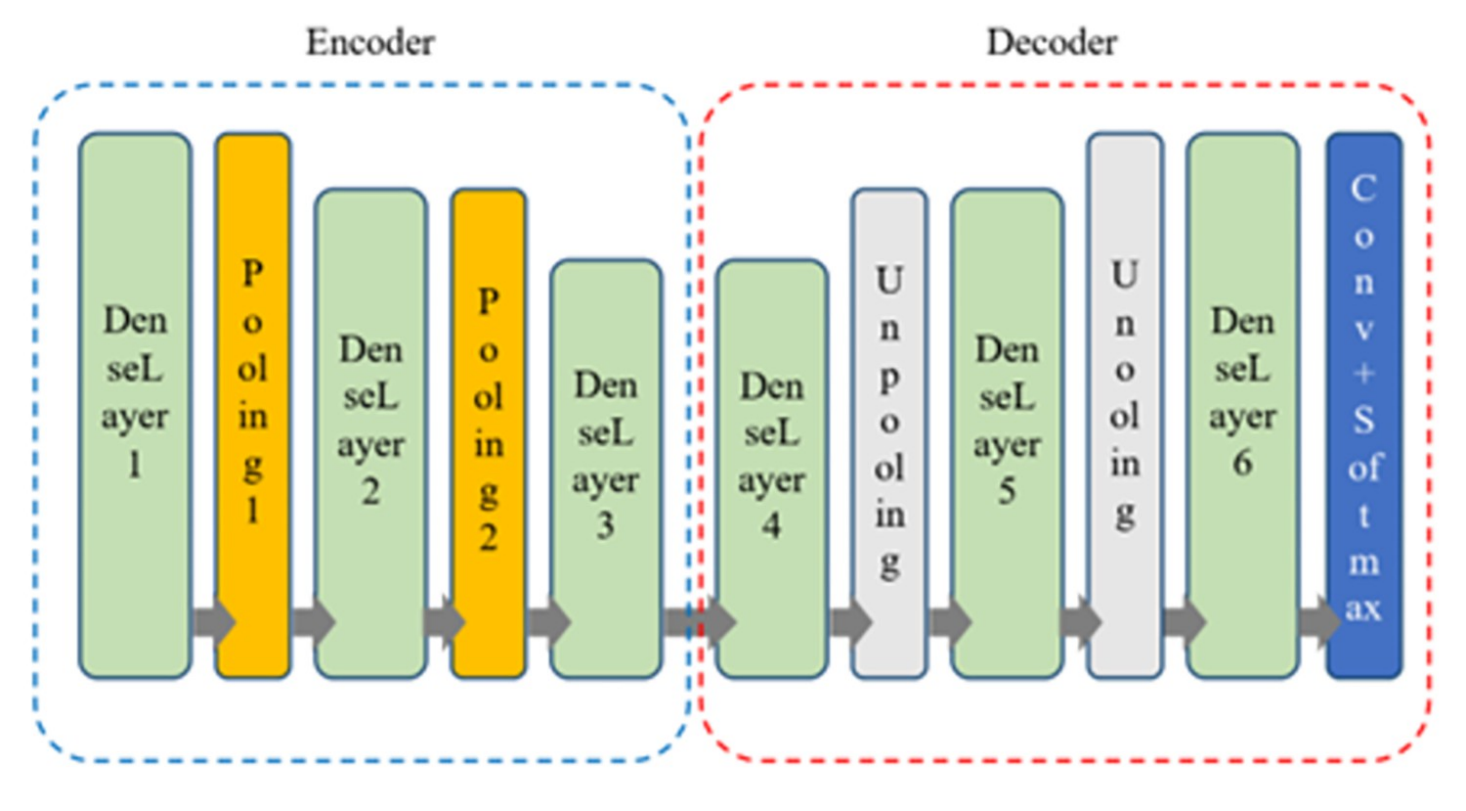
The basic CNN pattern used in the proposed method for NBIS.

Considering the described structure of dense layers, the basic CNN pattern in the proposed method has a structure according to Fig 3.

According to Fig 3, the proposed CNN model includes six dense layers, for which the structure of each layer is shown in Fig 2. In addition, this network contains four pooling layers as transfer functions and a Softmax layer to determine the segmentation output. Tunable hyperparameters in each convolution layer of dense layers are filter size, length, and width. These three parameters can also be tuned for the last layer of the network. On the other hand, each pooling layer can choose one of the max or average functions. Thus, the number of tunable hyperparameters in the proposed model equals 59. These hyperparameters are the number, length, and width of 19 convolution layers in the network and two parameters related to the type of pooling function in the encoder part of the network. All these parameters are determined by a set of LAs and explained in the next section.

### 2.2. Configuration of CNN model based on LA

**Fig 4:**
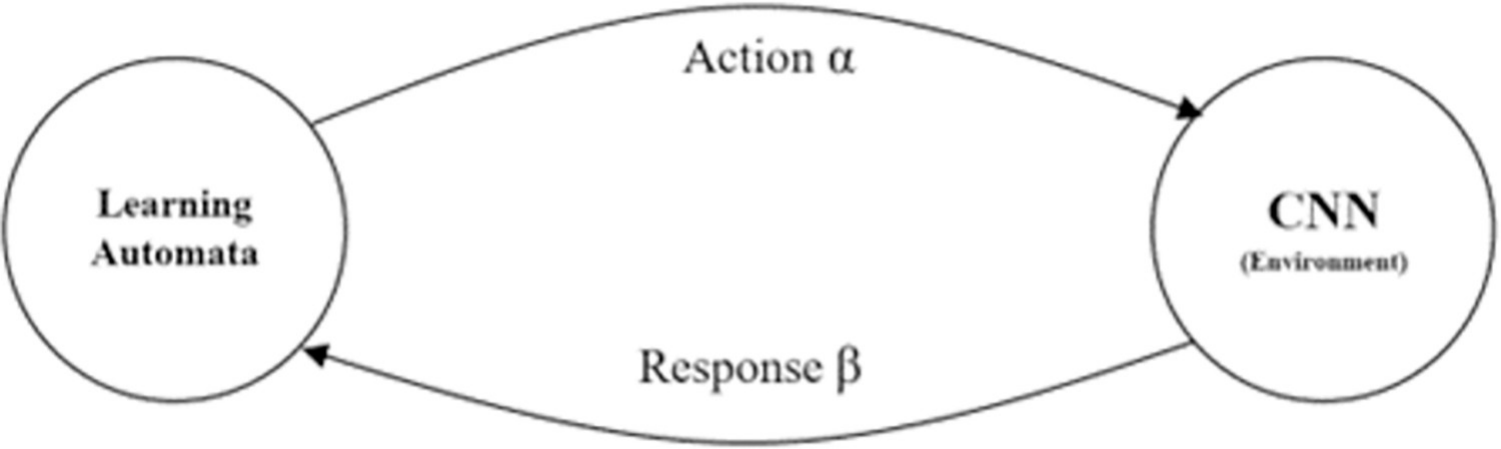
LA structure used in the proposed method.

As mentioned, the proposed method utilizes an RL strategy to optimally configure the CNN structure, and includes a set of LA models, which use a simple learning mechanism. This structure is shown in Fig 4.

As mentioned in Section 1, the LA model is used to dynamically adjust the hyperparameters of the CNN to optimize its configuration for neonatal brain image segmentation, and adaptively find the best architecture and parameters, such as filter sizes, pooling types, and number of layers, by employing a reward and penalty mechanism during the training process. LA has a set of selectable actions. This set is described as *A* = {*α*_1_, *α*_2_,…,*α*_*n*_}. Each action in set A has a selection probability. In the proposed method, the selection is done randomly in 30% of repetitions and is based on the probability vector of automata in the other repetitions. Each LA operates by selecting an action from set A and applying it to the CNN structure. By repeating this process with all LA models in each cycle, a candidate configuration for the CNN model is created. This candidate model is trained using database training samples, and its segmentation quality is evaluated using DICE criteria. In this case, the quality evaluated for segmentation by the candidate CNN model is considered as the environment’s reaction. Then, each LA uses the environment’s response to update its probability vector and choose the following action. During this process, each LA learns which configuration mode is optimal for network layers by tuning the probability of actions based on reward and penalty parameters. In this way, an LA is assigned to each configurable layer (convolution and pooling layers) in the proposed CNN model. Considering the presence of 6 dense layers in the proposed CNN model, each containing three convolution layers, the number of convolution operators in these layers is equal to 18, and including the last layer (Softmax), this number is 19. In this way, an LA is assigned to each of these layers, to determine the hyperparameters for the corresponding layer, which are filter number, length, and width. It must be noted that:

- number of filters has 15 possible states and is multiple of 16 in the range of 32 to 256.
- Filter length has five possible states and is an odd number from 1 to 9.
- Filter width has five possible states in the range of 1 to 9.

According to the values defined for the above hyperparameters, the number of unique states that can be selected for each layer is equal to 15×5×5 = 375, and the task of the LA assigned to each convolution layer is to determine the optimal configuration among these 375 states or actions. Each action is described as 〈*N*_*i*_, *L*_*i*_, *W*_*i*_〉 whose members represent the number, length, and width of the filter, respectively. At the moment of formation of the LA structure for each convolution layer, all actions have the same probability of 1/375. The second class of LAs is assigned to the pooling layers in the network coding section. In this case, two LA with two selectable actions are assigned to the pooling1 and pooling2 layers (see Fig 3). Selectable actions in these LAs are the *MaxPool* transfer function and the *AveragePool* transfer function, which are described by numbers 0 and 1, respectively. It is obvious that in this case, the initial selection probability vector for each of the LA is {1/2, 1/2}.

The pseudocode related to RL-based hyperparameter tuning is presented below. This pseudocode clearly outlines the steps for selecting and updating hyperparameters based on LA model and DICE criterion.

**Step1:** Initialize CNN and LAs for each layer

• Define actions for each LA (e.g., filter size, number, pooling type).

• Initialization of action probabilities uniformly.

**Step2:** Set parameters: Max_Iterations, No_Improvement_Threshold, α (reward), β (penalty).

Step 3:

for I = 1: Max Iterations:

for LA in CNN:

• Select an action based on its probability.

• Configure CNN with selected actions.

• Train CNN on training data.

• Evaluate model performance using DICE and ASD metrics.

if performance improves:

Apply reward (α) to selected actions.

else:

Apply penalty (β) to selected actions.

if No_Improvement_Threshold is reached (consecutive iterations without improvement), TERMINATE.

**Step 4:** Output optimal CNN configuration.

With these explanations, the proposed RL model includes 21 LAs that work together iteratively for the optimal configuration of the CNN model. During each iteration, each LA first selects one of its actions and applies the chosen action to its corresponding layer in the basic CNN model. After applying the selected actions by all LAs, a configured CNN is formed. After that, this network is trained using database samples, and the efficiency of the trained model is evaluated using the DICE criterion. Then, according to the received result, the probability vectors of all the LAs are updated using reward and penalty operators. Thus, after receiving the environment’s response, the obtained quality value is compared with the highest value obtained in the previous iterations, and according to the result of this comparison, the probabilities of each LA model are updated, in which the following conditions may occur:

- If the quality of the current iteration is greater than the highest quality in the previous iterations for the currently determined configuration, the set of actions selected by LAs in this cycle is considered optimal choices. In this case, each LA increases the probability of choosing the latest action using the following relationship (current action *i* is considered) [21]:

$$p_{j}(k+1)=\left\{ \begin{array}{l} p_{j}(k)+a[1-p_{j}(k)]\;j=i,\\ (1-a)p_{j}(k)\;\forall j\neq i. \end{array}\right.$$ (2)

in which, a and b are reward and penalty coefficients, respectively. In the proposed method, these two parameters are considered equal to 0.5. Also, k is the number of times the probabilities are modified.

- If the quality of the current iteration is lower than the highest quality in the previous iterations, the response produced in the last cycle (and the hyperparameters selected by the LAs) are considered non-optimal choices. In this case, each LA reduces the probability of choosing the previous action using the following relationship [25]:

$$p_{j}(k+1)=\left\{ \begin{array}{l} (1-b)p_{j}(k)\;j=i,\\ (\frac{b}{K-1})+(1-b)p_{j}(k)\;\forall j\neq i. \end{array}\right.$$ (3)

in which, K represents the number of actions that can be selected in each LA (the number of possible combinations for the configuration of each layer). After applying the above conditions to each of the actions of the LAs (individually for each layer), the probability vectors of all the LAs are updated. After updating the LA models, the selection processes, environment response evaluation, and probability vector update are repeated from the first step. This process continues until one of the termination conditions is met.

## 3. Simulations, results, and discussions

In this section, the performance evaluation of the proposed method is discussed. The simulation was implemented in MATLAB 2020a software environment. All the tests in this research were performed on a desktop computer with Windows 10 64-bit operating system, Intel Core i7 3.2 GHz processor, and 8 GB of memory. The processing operations related to CNN were implemented through the Graphical Processing Unit (GPU) in the Nvidia 820 GTX graphics adapter with the ability to support Compute Unified Device Architecture (CUDA). To perform the experiments, the iSEG17 database was used [22], which consists of 23 de-identified T1 and T2 MRI images of six-month-old infants, created under strict ethical guidelines and anonymization protocols to ensure privacy. No personally identifiable information (PII) is included, and all scans are labeled with random identifiers. The dataset was collected with informed consent and adheres to IRB-approved protocols. All tests are conducted on secure servers with limited access, adhering to strict data privacy standards. The processed data and results are used exclusively for research purposes [22]. As a result, the privacy of the subjects involved in the iSeg-2017 dataset is fully preserved throughout the research. In this database, 10 training samples with target labels are used to build the learning model, and the remaining 13 samples, without labels, are used to evaluate the trained model. The dimensions of each sample are equal to 144×192×256, where each voxel represents 1 mm^3^. Labeling of database samples was done based on three target classes: White Matter (WM), Gray Matter (GM), and Cerebrospinal Fluid (CSF). Images were normalized to have a mean of 0 and a variance of 1 before being fed into the network.

The proposed model is a CNN network with an encoder-decoder architecture, comprising 6 dense layers, where each layer includes 3 convolutional layers for feature extraction, batch normalization to improve training speed and stability, and ReLU activation functions for non-linear output transformations. The filter dimensions and the type of pooling functions in each layer were optimized using the mentioned LA model. The reinforcement learning settings included equal initial probabilities for layer parameters and a reward and penalty set to 0.5. The learning process continued until reaching a maximum of 200 epochs or no improvement in the segmentation quality evaluation criteria after 15 consecutive epochs. During the experiments, the performance of the proposed method was evaluated based on the following criteria:

1. DICE score: This criterion determines the similarity of each region segmented by the algorithm to the actual state. This criterion describes the accuracy of the segmentation algorithm, and the goal of each method is to achieve higher values of the DICE score. A value of 1 for this criterion indicates complete accuracy in segmentation. This criterion can be calculated using the following relationship:

DICE=2|S∩T||S|+|T| (4)

where S represents the segmentation result, and T specifies the Ground-Truth segmentation for each sample. Also, |S| shows the size of the set S.

2. Average Surface Distance (ASD): This criterion describes the average difference between the boundaries of the segmented regions and the actual segmentation boundaries. Achieving a lower value of ASD indicates a more significant match of the segmentation result with the ground truth state, and a value of zero for this criterion indicates a complete match. This criterion can be calculated using the following relationship:

$$ASD(T,S)=\frac{1}{2}(\frac{\sum_{X_{i}\in R_{S}}\underset{X_{j}\in R_{T}}{\min}d(X_{i},X_{j})}{\sum_{X_{i}\in R_{S}}1}+\frac{\sum_{X_{j}\in R_{T}}\underset{X_{i}\in R_{S}}{\min}d(X_{i},X_{j})}{\sum_{X_{j}\in R_{T}}1})$$ (5)

where *R*_*S*_ and *R*_*T*_ represent the output and ground segmentation regions, respectively. Also *d*(*X*_*i*_,*X*_*j*_) shows the Euclidean distance between two points *X*_*i*_ and *X*_*j*_.

In configuring the CNN model with RL, the number of iterations of the algorithm was set to T = 200, and the maximum number of unchanged iterations was set to K = 15. Also, the reward and penalty parameters in all LAs were determined as a = 0.5 and b = 0.5, respectively. Each sample was defined as *B*×*M*×*H*×*W*×*L*, where *B* and *M* represent the size of the batch and the number of input modalities, respectively. Also, *H*, *W*, and *L* represent the sample size in sagittal, coronal, and axial dimensions, respectively. In the process of conducting experiments, each sample was considered as 8×2×32×32×32. In the following, the results of evaluating the proposed method based on this configuration are presented.

**Fig 5:**
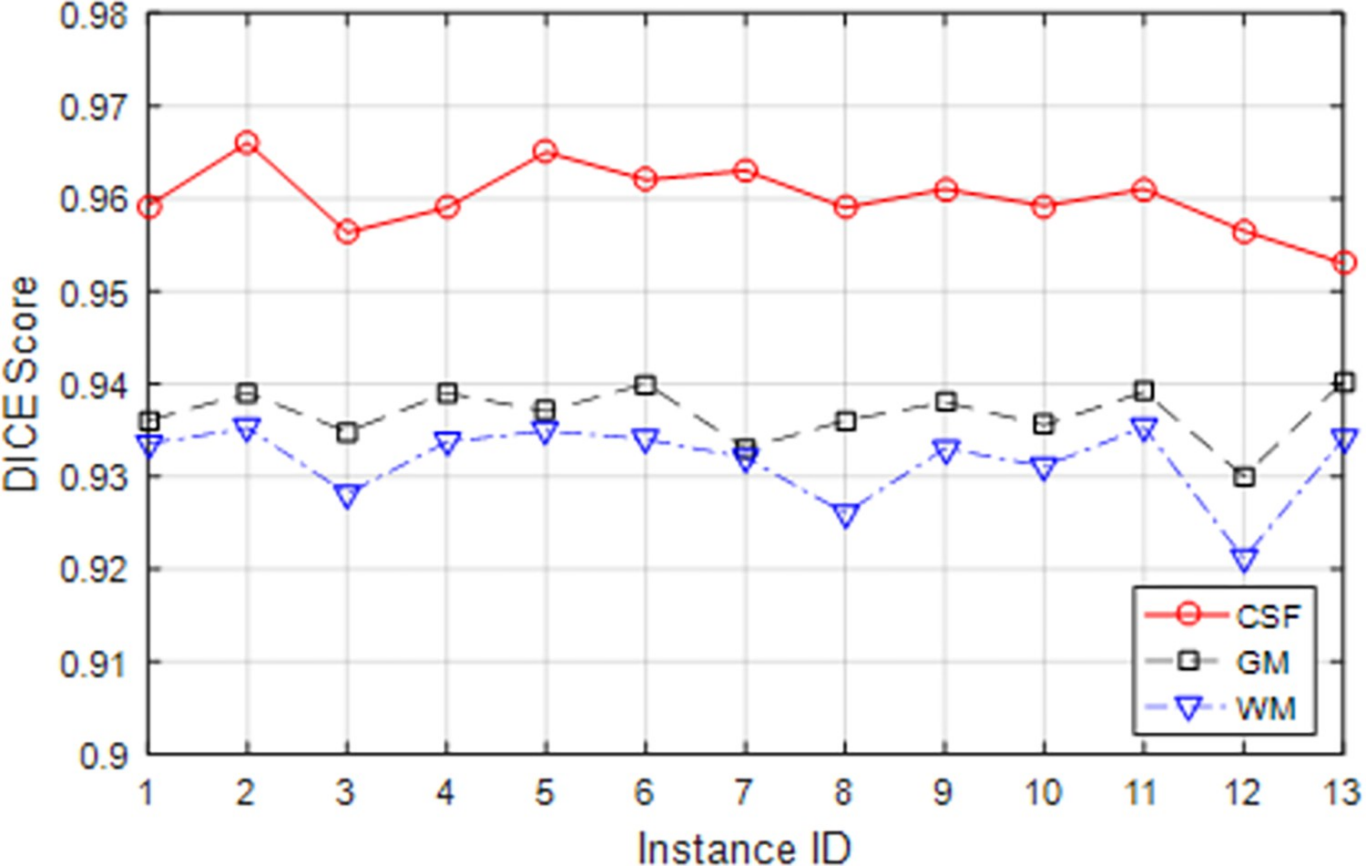
DICE score values resulting from segmentation by the proposed method for test samples.

Fig 5 shows the DICE score values for 13 database test samples for each image region separately. As the results show, segmentation can be done with high accuracy by using the proposed method. The general review of the results shows that the segmentation accuracy for the CSF region is more than that of the GM and WM regions. The reason for this can be attributed to the low contrast of NBIs. Because the low contrast of an MRI image causes the difference in brightness values in the GM and WM regions to decrease. For this reason, it isn’t easy to separate these regions. On the other hand, the CSF region appears as a darker region in T1 images and as a brighter region in T2 images; it has a more apparent difference with GM and WM regions. This feature has caused the accuracy of the proposed method to detect the CSF region more than the other two regions. Nevertheless, the results presented in this experiment showed that the proposed method can distinguish the regions of NBI with an accuracy of at least 92%, which shows a significant improvement in the performance of the proposed method compared to previous methods.

**Fig 6:**
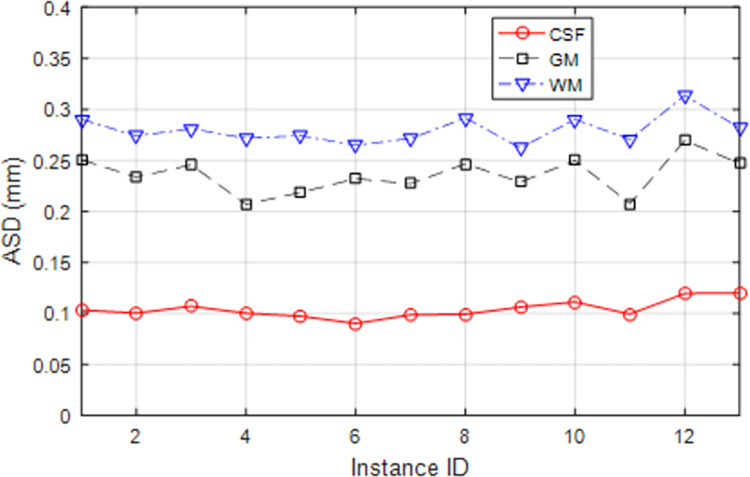
ASD values of the proposed method for test samples of the database.

Fig 6 shows the ASD values resulting from the segmentation of database test samples by the proposed method. In this diagram, the average distance values between the levels are drawn separately for each region. These results also confirm that the proposed method could detect the CSF region more accurately than others, and the lower ASD values for this region indicate a lower difference between the detected borders of the CSF region and the ground truth state.

The results of the DICE and ASD evaluation criteria show that the proposed method can identify the CSF, WM, and GM regions with an average accuracy of 96%, 93.17%, and 93.67%, respectively, and the average difference of the segmented borders for the CSF and WM regions, and GM with ground truth state is equal to 0.1039, 0.2797, and 0.2356 respectively.

**Fig 7:**
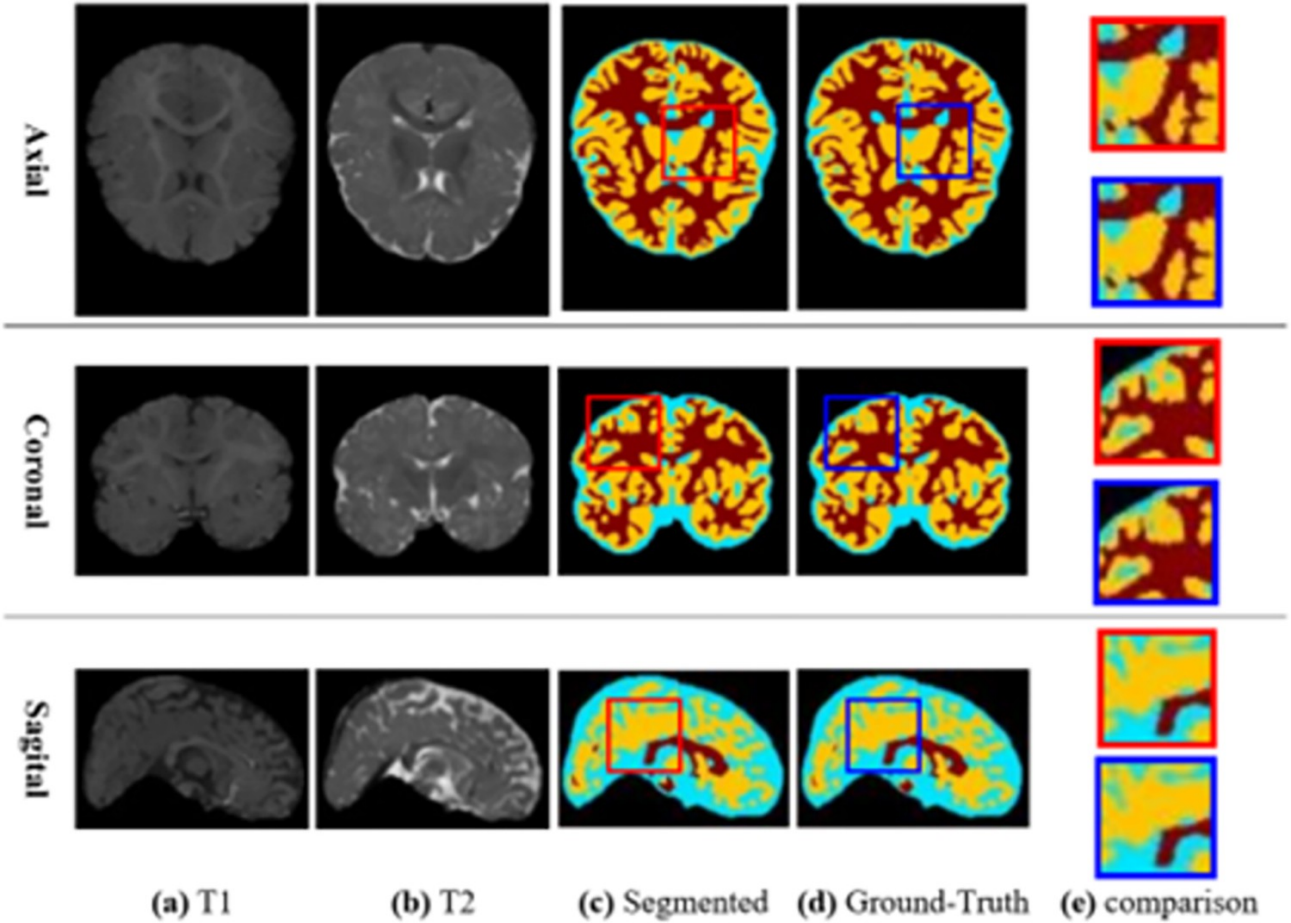
Segmentation results of one of the database images in different dimensions and ground-truth state.

Fig 7 shows the segmentation results of one of the database images. In the first row of this figure, the segmentation results for a slice of the sample image in the axial dimension are given. Also, in the second and third rows of this figure, segmentation results for cutting the sample in sagittal and coronal dimensions are drawn, respectively. In addition, columns (a) and (b) show the T1 and T2 images for the input sample, respectively. Column (c) shows the result of segmentation by the proposed method, and column (d) shows the ground truth segmentation of this image. Also, the comparison between the output of the proposed method and the ground truth state for a random image range is given in column (e). In this column, the zoomed range corresponding to the output of the proposed method is depicted on the left side, and the zoomed range corresponding to the ground truth state is depicted on the right side.

Examining the different regions of the segmentation result and matching it with the ground truth state shows that the output of the proposed method has a high correspondence with the ground truth state, and it confirms the high accuracy of the proposed method in NMIS. This high accuracy in the proposed method can be attributed using RL strategy to determine the optimal configuration of the CNN model. In the proposed method, the set of LAs tries to create a configuration with the highest accuracy for the CNN model by tuning hyperparameters related to its layers during iteration-based cycles.

**Fig 8:**
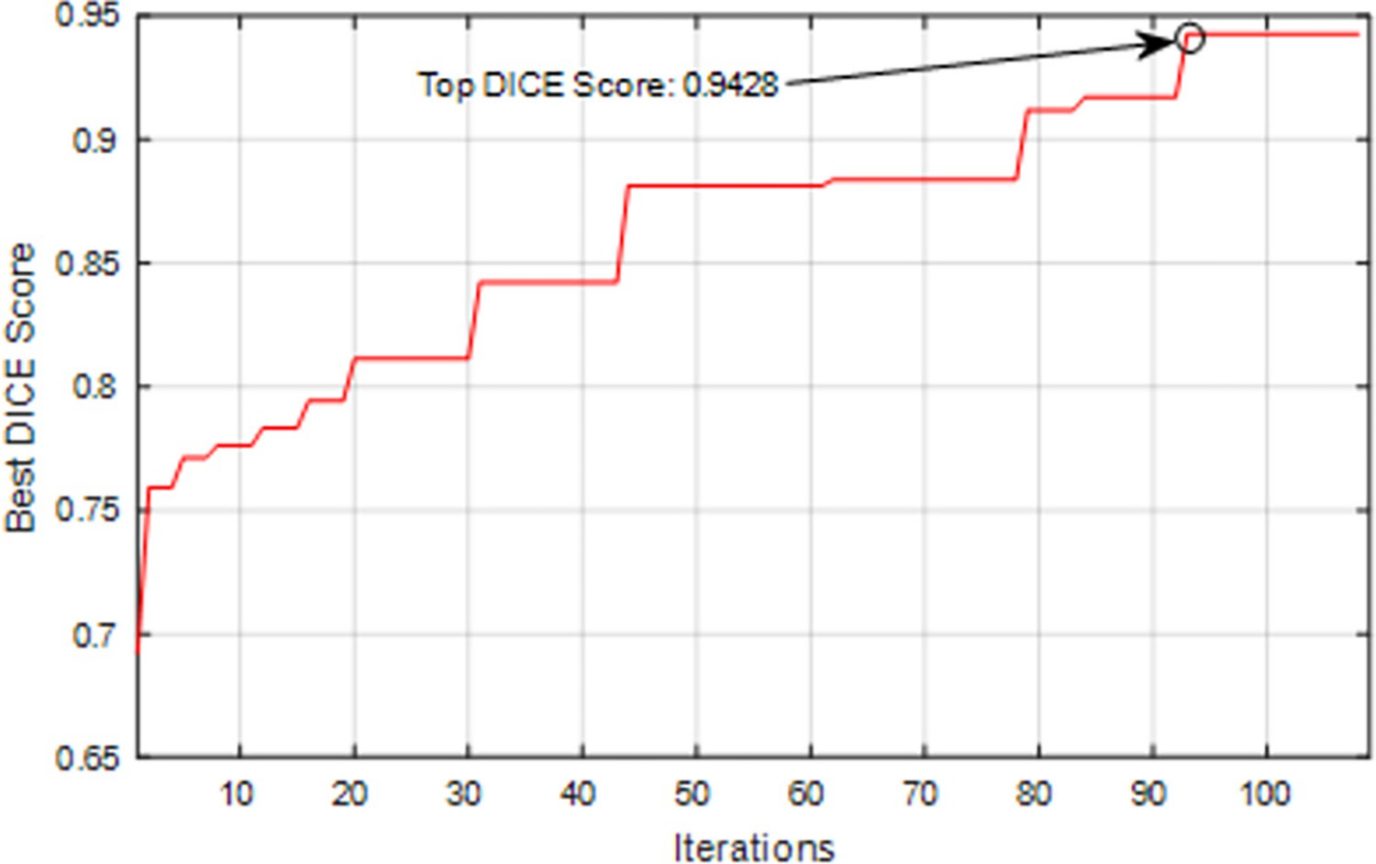
Best DICE score obtained for CNN model during different configuration iterations by LAs.

Fig 8 shows the accuracy changes (DICE score) obtained for the CNN model during different iterations of the proposed algorithm. In this diagram, the horizontal axis shows the iterations of the proposed algorithm, and the vertical axis shows the accuracy value obtained for the best configuration during different cycles. Based on the results presented in this diagram, LA models can maximize segmentation accuracy by progressing through algorithm iterations. The DICE value displayed in this graph is the average value of the CSF, WM, and GM regions. This diagram shows that after 108 iterations, the configuration process of the CNN model is terminated. Because after the 93rd cycle, there is no improvement in the accuracy of the neural network, and in other words, the set of LAs cannot create a more suitable sequence for the CNN model. Therefore, considering that the threshold of the number of iterations without improvement is K = 15, the algorithm ends in the 108^th^ cycle.

In Tables 1 and 2, the efficiency of the proposed method in the correct segmentation of target regions is compared with some previous methods. In the study, a wide range of division methods to compare with the proposed approach are based on their prominence and effectiveness in previous research. The methods selected for comparison are APRNet [13], Nonlocal U-Net [14], FC-Semi-DenseNet1 [15] and 2 [20], and DAL-AL [9] and Msl-Skku [12]. These algorithms are widely recognized in the literature for their performance in image segmentation tasks, especially in the field of medical imaging. APRNet is known for its ability to employ attention mechanisms, which enhance feature representation. Previous studies have demonstrated its effectiveness in various segmentation tasks, providing a solid baseline for comparison. Non-local U-Net algorithm involves non-local operations and has shown improved segmentation performance in scenarios where global context is critical. Its inclusion allows us to evaluate how our method performs against an architecture designed for spatial context. FC-Semi-DenseNet1 and 2 methods use dense connections to facilitate feature reuse and increase gradient flow during training. They are noted for their strong performance in segmentation challenges, making them suitable benchmarks. DAL-AL uses adaptive learning strategies and has shown strong performance in segmentation tasks. Msl-Skku has also shown superior performance in the MICCAI Grand Challenge in MRI segmentation of 6-month-old infants’ brain. Its performance measures serve as a valuable reference point for evaluating the improvements provided by our method. The effectiveness of these comparison methods is summarized in Tables 1 and 2 using two key metrics: the DICE score and the ASD.

**Table 1:** Effectiveness comparison of the proposed method in segmentation of iSeg17 test samples with previous methods based on DICE score.

Algorithm	DICE Score			
	CSF	GM	WM	AVG.
**APRNet [13]**	0.955	0.924	0.911	0.93
**Non-local U-Net [14]**	0.953	0.9245	0.9103	0.9229
**FC-Semi-DenseNet1 [15]**	0.96	0.92	0.9	0.9267
**FC-Semi-DenseNet2 [15]**	0.96	0.92	0.9	0.9267
**DAL-AL [9]**	0.9568	0.9349	0.926	0.9392
**Msl-Skku [12]**	0.959	0.921	0.908	0.93
**Proposed Method**	**0.96**	**0.9367**	**0.9317**	**0.9428**

**Table 2:** Effectiveness comparison of the proposed method in segmentation of iSeg17 test samples with the previous method based on ASD values.

Algorithm	ASD (mm)			
	CSF	GM	WM	AVG.
**APRNet [13]**	0.12	0.32	0.35	0.26
**Non-local U-Net [14]**	0.14	0.37	0.4	0.3
**FC-Semi-DenseNet1 [15]**	0.14	0.35	0.38	0.29
**FC-Semi-DenseNet2 [15]**	0.12	0.34	0.41	0.29
**DAL-AL [9]**	0.11	0.25	0.28	0.21
**Msl-Skku [12]**	**0.109**	**0.315**	**0.368**	0.264
**Proposed Method**	**0.1039**	**0.2356**	**0.2797**	**0.2064**

The DICE coefficient is a critical measure to evaluate the overlap between the predicted segmentation and the ground truth. Our proposed method achieves a DICE score of 0.9428, which outperforms all compared methods, indicating a superior ability to capture related structures in test samples. The average symmetric distance indicates the accuracy of the segmentation boundary. The proposed method also shows an ASD of 0.2064 mm, which is the lowest value among the compared algorithms, and shows its capacity for accurate boundary delineation. These results show that dynamic evaluation of different combinations of hyperparameters in our CNN model by RL strategy has been effective in improving NBIS quality, compared to other methods that a static configuration is used.

### 3.1. Computational overhead and network complexity of the proposed algorithm

The primary source of computational overhead arises from the iterative process of configuring the CNN using LA model. As mentioned before, each LA is responsible for tuning multiple hyperparameters across the network layers, which involves repeated evaluations and updates based on the DICE and ASD segmentation quality metrics. Although this iterative approach increases computational time, it significantly enhances segmentation accuracy, as demonstrated in our results. To quantify the trade-off, we compared the proposed method’s accuracy with the computational time required for different configurations. Our experiments show that while simpler configurations result in faster processing, they achieve lower segmentation quality, particularly in challenging regions such as Gray Matter (GM) and White Matter (WM). The trade-off analysis indicates that the optimal configuration, which achieves an average DICE score of 94.28% and the lowest ASD value of 0.1039 mm, involves a reasonable increase in computation time (approximately 25% longer) compared to the fastest configuration. This demonstrates that our method strikes a balance between accuracy and efficiency by avoiding overly complex configurations that do not contribute significantly to performance improvement. Furthermore, the parallel implementation of CNN training on a GPU platform mitigates the computational overhead, allowing for efficient execution even with complex network architectures. This parallelization ensures that the method remains viable for real-world applications without sacrificing segmentation quality.

The proposed LA approach effectively tackles increasing network complexity by dynamically selecting optimal hyperparameters (e.g., filter size, pooling types) using reinforcement learning principles. This enables CNN to adapt its structure based on the segmentation task’s complexity without manual tuning. As shown in [23], managing complexity in deep networks often involves dynamically adjusting the network depth and layer configurations. The LA model in our method achieves similar outcomes by iteratively optimizing hyperparameters, ensuring that the network maintains high performance without unnecessary growth. As well as in [24], reinforcement learning is used to balance the trade-off between network accuracy and computational load. Our approach aligns with this concept by using reward and penalty mechanisms to converge on efficient configurations that limit unnecessary increases in network size. Study [25] also shows that demonstrates how adaptive mechanisms can improve segmentation quality in complex medical image scenarios. Our method similarly uses LA models to ensure that network complexity scales appropriately with the segmentation task’s difficulty, achieving optimal segmentation quality even with increasing network depth and layer count. These strategies collectively help our proposed method manage network complexity efficiently, resulting in a robust and adaptive segmentation model for neonatal brain images. It is worth noting that the performance of the proposed neural network model is influenced by factors such as network architecture, the number of layers, and learning parameters. While increasing the number of layers can enhance accuracy, it also heightens the risk of overfitting and demands more training data. Furthermore, optimizing learning parameters requires significant time and computational resources. Implementing these models typically necessitates powerful hardware like GPUs or TPUs, which may encounter memory limitations or long training times. Additionally, ensuring the model’s effectiveness in real-world scenarios and its adaptability to diverse medical images poses further challenges. Addressing these issues is essential for achieving optimal performance, particularly in medical applications.

## 4. Conclusion

In this article, a new method is presented for NBIS using a combination of deep learning and RL techniques. The proposed method includes a basic CNN model, whose hyperparameters of each layer are determined by LA models. In this method, an LA is assigned to each configurable layer of CNN, and these learning models work together iteratively to create a configuration with the highest level of segmentation accuracy and ensure continuous improvement of neural network performance in image segmentation. The efficiency of the proposed method was evaluated by iSeg17 database samples. The results show that the proposed method can segment the regions of NBI with an accuracy of at least 93%, which shows a significant improvement in the performance of the proposed method compared to previous ones. Efficiency comparison of the proposed method with previous ones confirms that dynamic configuration based on RL in the proposed method can be effective in increasing segmentation accuracy and reducing the difference of the output regions with the ground truth state.

One of the limitations of the current research is the need to manually determine the parameters of LAs, including reward and penalty parameters, as well as selectable value ranges for hyperparameters in CNN layers. Future work could incorporate state-of-the-art RL algorithms such as Deep Q-Networks (DQN) and Proximal Policy Optimization (PPO). These models are capable of handling high-dimensional action spaces and complex state representations, making them suitable for optimizing more complex CNN architectures. PPO, on the other hand, offers more stable policy updates, ensuring reliable convergence when tuning large networks with diverse configurations. Another promising direction is the application of multi-agent RL to optimize different components of the CNN architecture simultaneously. An exciting area for future research is the use of meta-RL to improve the adaptability and generalizability of the proposed approach. By training a meta-RL agent that learns a strategy for optimizing hyperparameters across multiple tasks or datasets, the system can be made to adapt quickly to new medical imaging scenarios without the need for retraining from scratch. Therefore, investigating these cases can be the subject of future research.
